# Association between the β-blocker use and patients with sepsis: a cohort study

**DOI:** 10.3389/fmed.2023.1272871

**Published:** 2023-10-26

**Authors:** Qilin Yang, Tianyu Kong, Ziping Bao, Shanshan Yang, Xiaohua Chen, Jiezhao Zheng, Xuming Xiong, Deliang Wen, Zhenhui Zhang

**Affiliations:** Department of Critical Care, The Second Affiliated Hospital of Guangzhou Medical University, Guangzhou, China

**Keywords:** β-blocker, sepsis, in-hospital mortality, critically ill patients, septic shock

## Abstract

**Objective:**

This study aimed to assess whether β-blockers are associated with mortality in patients with sepsis.

**Method:**

We conducted a retrospective cohort study of patients with sepsis using the Medical Information Market for Intensive Care (MIMIC)-IV and the emergency intensive care unit (eICU) databases. The primary outcome was the in-hospital mortality rate. The propensity score matching (PSM) method was adopted to reduce confounder bias. Subgroup and sensitivity analyses were performed to test the stability of the conclusions.

**Results:**

We included a total of 61,751 patients with sepsis, with an overall in-hospital mortality rate of 15.3% in MIMIC-IV and 13.6% in eICU. The inverse probability-weighting model showed that in-hospital mortality was significantly lower in the β-blockers group than in the non-β-blockers group [HR = 0.71, 95% CI: 0.66–0.75, *p* < 0.001 in MIMIC-IV, and HR = 0.48, 95% CI: 0.45–0.52, *p* < 0.001 in eICU]. In subgroups grouped according to sex, age, heart rate, APSIII, septic shock, and admission years, the results did not change.

**Conclusion:**

β-blocker use is associated with lower in-hospital mortality in patients with sepsis, further randomized trials are required to confirm this association.

## Background

Sepsis, a dysregulated immune response caused by infection, is a common syndrome in the ICU (1). However, despite medical advances and the initiation of active treatment, as well as the treatment of underlying infections and support for failing organ function, sepsis still has a high mortality rate of 20%–30% (2).

Djillali et al. showed sympatho-adrenergic system activation and significantly elevated circulating catecholamine levels in patients with sepsis (3). In critically ill patients, transient activation of the sympathetic nervous system is beneficial; however, excessive activation is detrimental to the organism (4). In addition, tachycardia is an independent predictor of poor prognosis in patients with sepsis (5). β-blockers are common drugs for the treatment of cardiovascular diseases, including chronic heart failure (6, 7) and myocardial infarction (8, 9) occur by reducing sympathetic neural activity and controlling the heart rate. Therefore, we hypothesi**z**ed that β-blockers could inhibit sympathetic activation, reduce catecholamine release, and control tachycardia in sepsis patients, thereby improving the prognosis of sepsis patients.

Several studies have shown that the application of β-blockers in sepsis reduces heart rate, morbidity, and mortality (10–13), but there is still a lack of studies with large samples. Moreover, the application of β-blockers in sepsis may also lead to decreased cardiac output, microcirculation, and tissue perfusion, resulting in adverse events (14). Therefore, we conducted a retrospective study to determine the association between the use of β-blockers and mortality in patients with sepsis to better guide clinical management.

## Methods

### Data sources and setting

The two patient cohorts used in this study were drawn from two large public databases, the Medical Information in Intensive Care database (MIMIC-IV) and the eICU Collaborative Research database v2.0. The MIMIC-IV is a large clinical database containing comprehensive high-quality data on ICU inpatients at the Beth Israel Deaconess Medical Center between 2008 and 2019, to which we obtained access (certification number: 36508141). The eICU database covers 200,859 patients admitted to the intensive care units of 208 hospitals in the United States between 2014 and 2015.

### Study population

All patients in the MIMIC-IV and eICU databases were eligible for inclusion. Adults (older than or equal to 18 years) diagnosed with sepsis-3 were included in our study. The diagnostic criterion was an increase in the Sequential Organ Failure Assessment (SOFA) score of 2 points caused by a dysregulated host response to infection. For patients with repeated ICU admissions, only those admitted to the ICU for the first time were considered.

### β-blockers exposure

Exposure to β-blockers was defined as a prescription containing a β-blocker within 3 days of admission to the ICU. The β-blockers included “Acebutolol,” “Atenolol,” “Esmolol,” “Betaxolol,” “Bisoprolol,” “Metoprolol,” “Nadolol,” and “Propranolol.” The routes of administration of β-blocker include intravenous push and oral administration.

### Covariates

We included the following variables: heart rate (HR), mean arterial pressure (MAP), blood glucose, temperature, SpO2, hemoglobin, platelet, white blood cell (WBC), bicarbonate, blood urea nitrogen (BUN), chloride, creatinine, potassium, acute physiology score (APS) III, simplified acute physiology score (SAPS) II, sequential organ failure score (SOFA), Charlson Comorbidity Index, and comorbidities (including Peripheral vascular disease, rheumatic disease, peptic ulcer, diabetes, septic shock), use of β-blockers prior to ICU admission, use of statins during hospitalization and the use of ventilator and renal replacement therapy (RRT) on the first day of ICU admission. Basic information for hospital admission registration was obtained which included demographic characteristics (e.g., age and gender).

### Outcome

The primary outcome was in-hospital mortality. The secondary outcomes included the length of ICU stay, length of hospital stay, ventilator-free days until 28 days, vasopressor-free days until 28 days, 28-day mortality, 90-day mortality, and 365-day mortality.

### Statistical analysis

Continuous variables were expressed as the mean ± standard deviation (SD) or median and interquartile range (IQR), and categorical variables were presented as frequency (percentage). For continuous variables, the Student’s t-test or Wilcoxon rank-sum test was used to determine whether they conformed to normal distributions. Pearson’s chi-squared test or Fisher’s exact test was used for categorical data, as appropriate.

Multivariable Cox regression analyses were used to assess the independent association between-blocker use and in-hospital mortality and to calculate hazard ratios (HRs) and their 95% confidence intervals (CIs). Survival curves were generated using Kaplan–Meier analysis and compared by log-rank analysis. To balance the baseline characteristics between the -blocker and β-blocker use groups, we used the PSM method, which was performed in our study by a 1:1 nearest neighbor matching algorithm with a caliper width of 0.2. The variables shown in Table 1 were all selected to generate the propensity score, and the standardized mean difference (SMD) was used to examine the degree of PSM. Less than 0.1 was considered an acceptable threshold. The primary outcome was further verified using inverse probability of treatment weighted (IPTW), which was created using the estimated propensity scores as weights. Subgroup analyses were stratified according to relevant covariates to reduce the impact of survival bias.

**Table 1:** Baseline characteristics of the included patients in the MIMIC-IV database.

Patient characteristic	Unmatched Patients			SMD	Propensity-Score–Matched Patients			SMD
	Total (*n* = 23,828)	Non-β-blockers (*n* = 13,390)	β-blockers (*n* = 10,438)		Total (*n* = 15,718)	Non-β-blockers (*n* = 7,859)	β-blockers (*n* = 7,859)	
Male, *n* (%)	13,763 (57.8)	7,383 (55.1)	6,380 (61.1)	0.12	9,166 (58.3)	4,578 (58.3)	4,588 (58.4)	<0.01
Age(years)	65.1 ± 16.4	62.1 ± 17.7	68.94 ± 13.6	0.44	67.4 ± 15.0	67.5 ± 15.9	67.3 ± 14.0	0.01
**Vital signs**								
Heart rate(bpm)	86.8 ± 16.0	87.0 ± 16.7	86.6 ± 15.1	0.02	86.5 ± 15.8	86.4 ± 16.1	86.5 ± 15.4	<0.01
MAP(mmHg)	76.7 ± 10.2	76.3 ± 10.6	77.1 ± 9.6	0.09	77.1 ± 10.3	77.1 ± 10.9	77.2 ± 9.8	0.01
Temperature(°C)	37.4 (37.0, 37.9)	37.5 (0.9)	37.5 (0.7)	0.10	37.4 (37.0, 37.9)	37.4 (37.0, 37.9)	37.4 (37.0, 37.8)	<0.01
Spo2(%)	93.0 (90.0, 95.0)	90.9 (7.9)	91.8 (5.9)	0.12	93.0 (90.0, 95.0)	93.0 (90.0, 95.0)	93.0 (90.0, 95.0)	0.01
**Laboratory tests**								
Glucose(mg/dL)	143.9 ± 47.6	145.6 ± 52.0	141.7 ± 41.2	0.08	144.2 ± 46.5	144.3 ± 48.9	144.0 ± 43.9	0.02
Hemoglobin(g/L)	9.9 ± 2.1	9.9 ± 2.3	9.9 ± 2.0	0.04	10.0 ± 2.1	10.0 ± 2.2	10.0 ± 2.1	0.02
Platelets(×10^12^)	159.0 (110.0, 223.0)	178.9 (100.9)	172.3(88.9)	0.07	162.0 (114.0, 225.0)	165.0 (114.0, 226.5)	159.0 (114.0, 222.0)	<0.01
WBC(×10^9^)	13.8 (9.9, 18.7)	15.3 (8.4)	15.0 (7.0)	0.04	13.7 (9.8, 18.4)	13.4 (9.5, 18.4)	13.9 (10.1, 18.3)	0.01
BUN(mg/dL)	22.0 (15.0, 37.0)	32.0 (24.5)	27.9 (20.1)	0.18	23.0 (16.0, 36.0)	23.0 (16.0, 37.0)	22.0 (15.5, 35.0)	0.02
Creatinine(mg/dL)	1.1 (0.8, 1.8)	1.8 (1.6)	1.5 (1.3)	0.19	1.1 (0.8, 1.7)	1.2 (0.8, 1.7)	1.1 (0.8, 1.7)	0.02
Bicarbonate(mmol/L)	22.0 (18.0, 24.0)	20.5 (5.3)	22.0 (4.2)	0.31	22.0 (19.0, 24.0)	22.0 (19.0, 24.0)	22.0 (19.0, 24.0)	0.02
Potassium(mmol/L)	4.6 ± 0.8	4.6 ± 0.9	4.6 ± 0.7	0.02	4.6 ± 0.8	4.6 ± 0.9	4.6 ± 0.8	<0.01
Chloride(mmol/L)	102.2 ± 6.6	101.7 ± 7.2	103.0 ± 5.8	0.20	102.5 ± 6.3	102.5 ± 6.6	102.5 ± 6.0	<0.01
**Comorbidity disease, *n*(%)**								
Peripheral vascular disease	2,847 (11.9)	1,174 (8.8)	1,673 (16.0)	0.22	1,905 (12.1)	956 (12.2)	949 (12.1)	0.01
Rheumatic disease	861 (3.6)	446 (3.3)	415 (4.0)	0.03	601 (3.8)	299 (3.8)	302 (3.8)	0.01
Peptic ulcer disease	725 (3.0)	502 (3.7)	223 (2.1)	0.10	422 (2.7)	215 (2.7)	207 (2.6)	0.01
Diabetes	7,132 (29.9)	3,610 (27.0)	3,522 (33.7)	0.15	4,947 (31.5)	2,478 (31.5)	2,469 (31.4)	0.01
Statin use	3,211 (13.5)	1,379 (10.3)	1,832 (17.6)	0.21	2,226 (14.2)	1,103 (14)	1,123 (14.3)	0.01
Septic shock	10,090 (42.3)	5,314 (39.7)	4,776 (45.8)	0.12	6,275 (39.9)	3,155 (40.1)	3,120 (39.7)	0.01
**Severity of illness**								
Charlson comorbidity index	6.0 (4.0, 8.0)	5.6 (3.1)	6.0 (2.7)	0.15	6.0 ± 2.8	6.0 ± 2.9	6.0 ± 2.8	0.01
SOFA score	5.0 (3.0, 7.0)	6.0 (3.6)	5.2 (2.7)	0.23	5.0 (3.0, 7.0)	5.0 (3.0, 7.0)	5.0 (3.0, 7.0)	0.02
SAPS II	39.5 ± 14.5	40.0 (15.6)	38.9 (12.8)	0.08	39.4 ± 13.7	39.5 ± 14.0	39.3 ± 13.5	0.02
APS III	49.0 (36.0, 69.0)	58.3 (27.1)	50.2 (22.8)	0.32	48.0 (36.0, 66.0)	49.0 (36.0, 66.0)	48.0 (35.0, 67.0)	0.02
**Preadmission medication, *n*(%)**								
β-blockers	1,820 (7.6)	669 (5)	1,151 (11)	0.22	1,140 (7.3)	565 (7.2)	575 (7.3)	0.01
**Interventions**								
RRT(day1)	1,060 (4.4)	756 (5.6)	304 (2.9)	0.14	556 (3.5)	283 (3.6)	273 (3.5)	0.01
Ventilator(day1)	12,185 (51.1)	6,746 (50.4)	5,439 (52.1)	0.04	7,759 (49.4)	3,873 (49.3)	3,886 (49.4)	<0.01

Bpm, beats per minute; MAP, mean arterial pressure; WBC, white blood cell; BUN, blood urea nitrogen; SOFA score, sequential organ failure score; SAPS III, simplified acute physiology score; APS III, Acute Physiology Score; RRT, renal replacement therapy.

A two-tailed test was performed and *p* < 0.05 was considered statistically significant. All analyses were performed with R 3.3.2 (http://www.R-project.org, The R Foundation).

## Results

### Population

**Figure 1:**
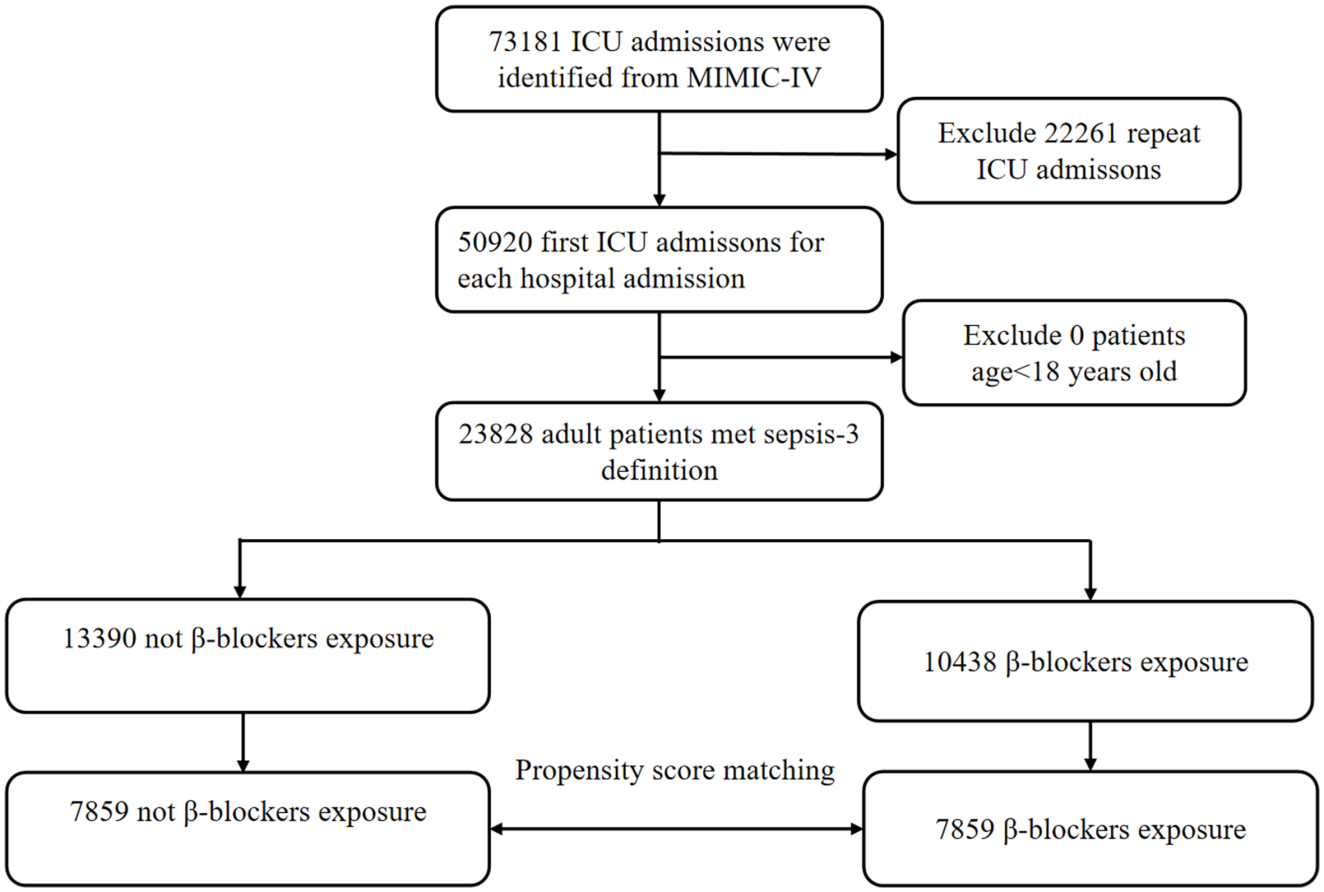
The flow chart of the study.

In the MIMIC-IV database, a total of 23,828 patients with sepsis were enrolled in our cohort, of whom 10,438 patients (43.8%) were exposed to β-blockers, and 13,390 (56.2%) were not (Figure 1). After PSM, 7,859 patients were successfully matched. In the eICU database, out of 200,859 unique patients, 37,923 patients were included in the study (Supplementary Figure 1).

### Study cohort and patients’ characteristics

The baseline patients’ characteristics are listed in Table 1. The baseline characteristics of the two groups after PSM are almost balanced in Table 1. The mean age was 65.1 ± 16.4 years old, 7,383 (55.1%) were males. The baseline characteristics of the included patients in eICU database are shown in Supplementary Table 1.

### Primary outcome

**Figure 2:**
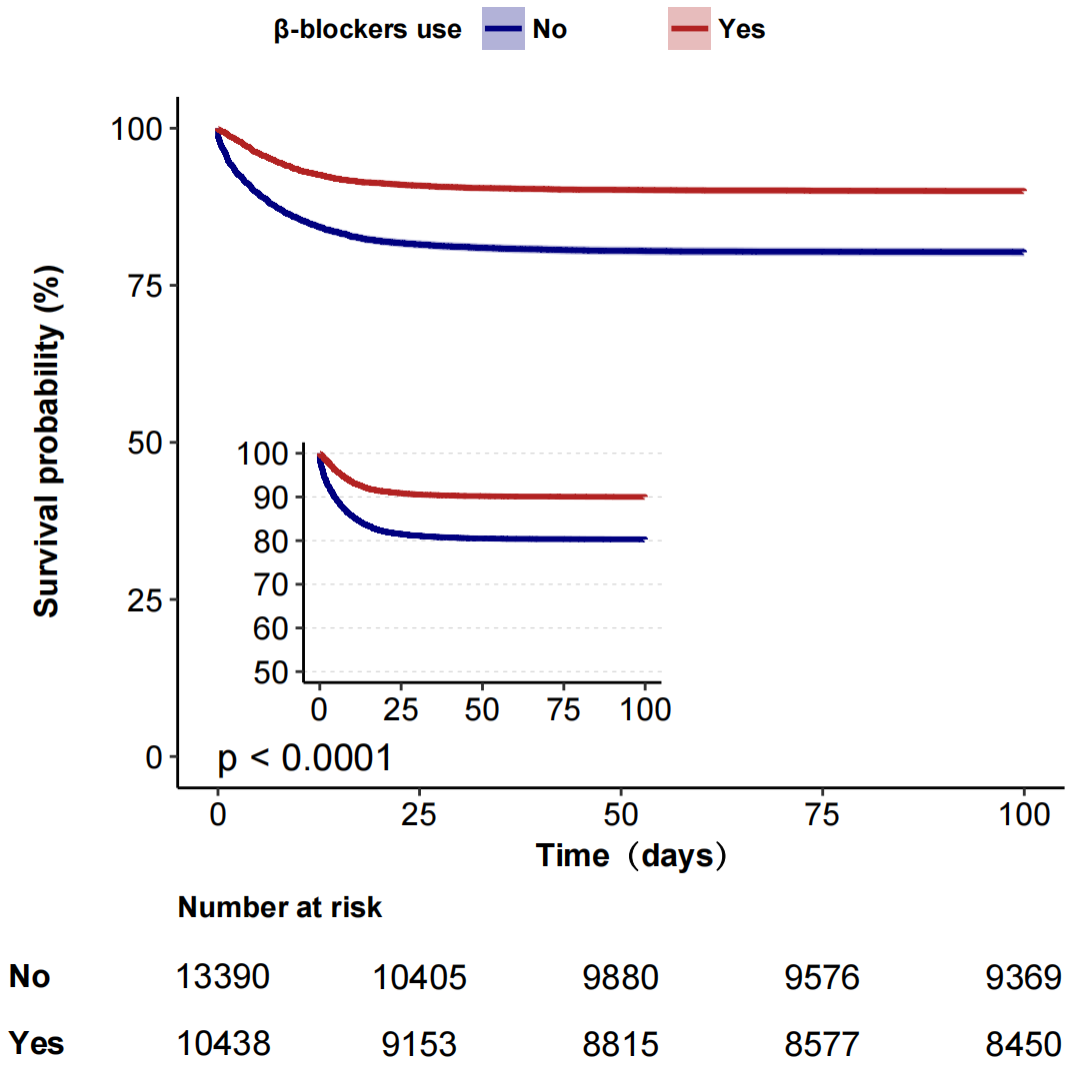
Kaplan–Meier estimates of cumulative probabilities of in-hospital survival for septic patients in β-blockers group and non-β-blockers group.

The overall in-hospital mortality rates were 15.3% in the MIMIC-IV group (Table 2) and 13.6% in the eICU (Supplementary Table 1). In the MIMIC-IV database, the in-hospital mortality for the β-blockers and non-β-blockers groups was 9.9%(1,032/10,438) and 19.5%(2,611/13,390), respectively. In univariable Cox proportional hazards regression, the HR was 0.47 (95% CI, 0.44–0.51, *p* < 0.001). In Multivariate Cox proportional hazards regression, the HR was 0.56 (95% CI, 0.52–0.61, *p* < 0.001). Similar to the results in the pre-matched cohort, both PSM (HR = 0.62, 95%CI, 0.57–0.67, *p* < 0.001) and IPTW (HR = 0.71,95% CI, 0.66–0.75, *p* < 0.001) indicated that β-blockers use was significantly associated with reduced in-hospital mortality. Before PSM, the in-hospital Kaplan–Meier survival curve also showed that β-blockers users had lower in-hospital mortality (Log-rank test: *p* < 0.0001; Figure 2). In the eICU database, we similarly observed that β-blockers were associated with reduced in-hospital mortality (adjusted HR 0.48, 95% CI, 0.45–0.52, *p* < 0.001; Supplementary Table 2).

**Table 2:** Association between β-blocker use and in-hospital mortality in the MIMIC-IV database.

Analysis	In-hospital mortality	*p*-value
**The primary outcomes**		
No. of events/no. of patients at risk(%)		
Total	3,643/23,828(15.3)	
Non-β-blockers	2,611/13,390 (19.5)	
β-blockers	1,032/10,438(9.9)	
Crude analysis-hazard ratio(95%CI)	0.47 (0.44,0.51)	<0.001
Multivariable-hazard ratio(95%CI)^a^	0.56 (0.52,0.61)	<0.001
with matching^b^	0.62 (0.57,0.67)	<0.001
Adjust for propensity score^c^	0.65 (0.60,0.70)	<0.001
Weighted.IPTW^d^	0.71 (0.66,0.75)	<0.001

^a^Hazard ratio from the multivariable Cox proportional model adjusted for all covariates (Table 1). ^b^Hazard ratio from a multivariate Cox proportional hazards model with the same strata and covariates matched according to the propensity score. The analysis included 15,718 patients (7,859 who received beta-blockers and 7,859 who did not). ^c^Hazard ratio from a multivariable Cox proportional hazards model with the same strata and covariates, with additional adjustment for the propensity score. ^d^Primary analysis with a hazard ratio from the multivariable Cox proportional hazards model with the same strata and covariates with inverse probability weighting according to the propensity score.

### Subgroup analysis

**Figure 3:**
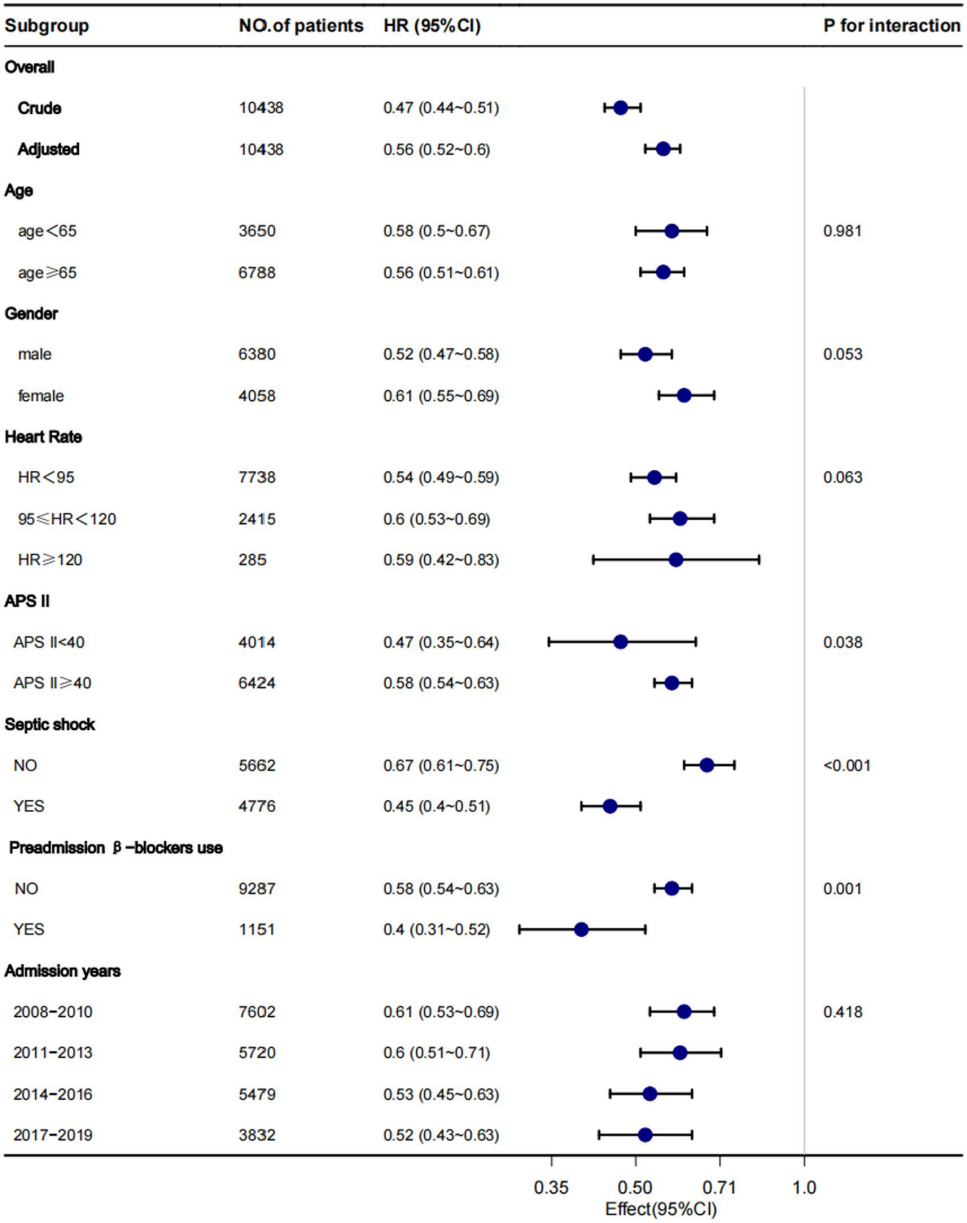
Subgroup analysis for the associations between β-blocker use and in-hospital mortality. Each stratification adjusted for all confounders (Table 1), except for the stratification factor itself.

Although subgroup analysis was performed according to some confounders, the in-hospital mortality survival analysis showed that patients in the β-blockers group had lower in-hospital mortality compared with the non-β-blockers in all the subgroups (Figure 3). However, some interactions were found between APS III, septic shock, and preadmission β-blockers use (*p* for interaction < 0.05).

### Sensitivity analysis

After we excluded patients with ICU stays of less than 24 h, acute myocardial infarction, and chronic heart failure, in-hospital mortality remained lower in the β-blocker patients than in the non-β-blocker patient group (all *p* < 0.001). Whether administered orally or intravenously, β-blockers were associated with reduced in-hospital mortality compared with the non-β-blockers group (Both *p* < 0.001; Table 3).

**Table 3:** Sensitivity analysis of the relationship between β-blockers use and in-hospital mortality.

Variable	No. of patients at risk (%)	Crude coefficient (95% CI)	Crude *p*-value	Adjusted coefficient^a^ (95% CI)	Adjusted *p-*value
Non-MI and Non-CHF	491 (8.6)	0.48 (0.44~0.53)	<0.001	0.6 (0.54~0.67)	<0.001
The length of ICU stay < 24 h	969 (10.1)	0.5 (0.46~0.54)	<0.001	0.59(0.55 ~0.64)	<0.001
Oral administration	567(7.4)	0.35 (0.32~0.38)	<0.001	0.46 (0.42~0.51)	<0.001
Intravenous administration	465(17)	0.85 (0.77~0.93)	0.001	0.75 (0.68~0.84)	<0.001

^a^Adjusted for confounders (Table 1). MI, acute myocardial infarction; CHF, chronic heart failure.

### Secondary outcomes

Table 4 shows secondary efficacy outcomes. Patients receiving β-blockers showed improved outcomes in terms of length of ICU stay, length of hospital stay, ventilator-free days until 28 days, vasopressor-free days until 28 days, 28-day mortality, 90-day mortality, 365-day mortality compared to those not receiving β-blockers.

**Table 4:** Secondary outcome analysis.

Variable	No. of patients	No. of events (%)	Crude coefficient (95% CI)	Crude *p*-value	Adjusted coefficient^a^ (95% CI)	Adjusted *p*-value
**Secondary outcomes**						
The length of hospital stay (d)	10,438		−2.04 (−2.35~−1.72)	<0.001	−1.04 (−1.36~−0.71)	<0.001
The length of ICU stay (d)	10,438		−1.05 (−1.22~−0.89)	<0.001	−0.49 (−0.65~−0.34)	<0.001
Vasopressor-free days until 28 days	10,438		2.89 (2.62~3.15)	<0.001	1.78 (1.53~2.03)	<0.001
Ventilator-free days until 28 days	10,438		3.1 (2.83~3.36)	<0.001	1.78 (1.54~2.03)	<0.001
28-day mortality	10,438	1,344 (12.9)	0.52 (0.49~0.55)	<0.001	0.59 (0.55~0.63)	<0.001
90-day mortality	10,438	1,946 (18.6)	0.58 (0.55~0.61)	<0.001	0.63 (0.59~0.67)	<0.001
365-day mortality	10,438	2,698 (25.8)	0.62 (0.59~0.64)	<0.001	0.65 (0.61~0.68)	<0.001

^a^Adjusted for all confounders (Table 1), except for the confounder itself.

## Discussion

In this large retrospective cohort study, we found that patients who received β-blockers after admission to the intensive care unit had a lower in-hospital mortality before and after removing the interference of possible confounding factors using PSM, compared with the non-β-blockers group. In the eICU database, we similarly observed that the β-blockers group was associated with reduced in-hospital mortality.

Morelli et al. conducted the first randomized controlled study to verify the efficacy and safety of esmolol in the treatment of septic shock. Akin to our findings, the results showed that the esmolol group had significantly lower 28-day mortality (49%) compared to 80.5% in the control group (adjusted hazard ratio, 0.39,95% CI,0.26 to 0.59, *p* < 0.001) (10). However, they only included patients with septic shock requiring vasopressors to maintain blood pressure, whereas we expanded the study population to include patients with sepsis (with and without vasopressors). Similarly, an observational study (*n* = 1,186) reported by Dmitri et al. showed that long-term β -blocker therapy (≥1 year) prior to hospital admission reduced 30-day all-cause mortality in patients with sepsis, but the study was conducted mainly in patients with sepsis on medicine wards, not in the ICU (11).

An RCT conducted by Kakihana et al. (*n* = 151) showed that landiolol significantly reduced heart rate and incidence of new-onset arrhythmia in patients with sepsis-related tachyarrhythmias. However, in their study, landiolol did not show a significant reduction in 28-day mortality in patients with sepsis (9% vs. 15%, *p* > 0.05), which is inconsistent with our findings, possibly because of the differences in sample size between the two studies and differences in patient demographics at baseline (e.g., patients in Kakihana’s study had higher SOFA scores, mean SOFA = 10.1) (15).

Interestingly, in our study, the mortality rate associated with sepsis was lower than that reported in previous studies (2), which may have been due to the use of different definitions of sepsis. However, in Rhee’s retrospective study of 173,690 septic patients with sepsis (according to the definition of sepsis-3) in the United States from 2009 to 2014, the in-hospital mortality rate of sepsis was 15.6%, which is comparable to our results (16), suggesting that our cohort was similar to those of previous studies.

One of the strengths of our study is that it included patients with sepsis from two large public databases (*n* = 62,676) and was the largest population included in any study that has simultaneously examined the relationship between β-blockers and sepsis.

In most studies, β-blockers are administered intravenously and rarely orally. In our study, the route of administration was both oral and intravenous, it was a real-world study combining the effects of β-blockers on sepsis mortality with oral as well as intravenous administration.

It has been labeled that β-blockers are protective in patients with cardiovascular disease (acute myocardial infarction, chronic heart failure), and in our study population, which included a subset of patients with comorbid cardiovascular disease (acute myocardial infarction, chronic heart failure), we were able to observe a survival benefit in patients receiving β-blockers even after excluding these patients.

However, the mechanism underlying the association between the use of β-blockers and lower mortality in patients with sepsis remains unclear. First, the protective effect of β-blockers in sepsis patients may be related to a lower heart rate and reduced sympathetic overexcitation in sepsis patients. In 1969, Berk et al. were the first to investigate the use to reduce sympathetic nerve excitability in sepsis, which is associated with improved survival (17). Second, it may be related to improved hemodynamics in patients with sepsis; Morelli et al. showed that esmolol can increase the stroke index, systemic vascular resistance, and left ventricular stroke work indices (10). Third, it may be related to the role of β-blockers in regulating inflammatory response. β-blockers can down-regulate inflammatory mediators (e.g., TNF-α, IL-6, HMGB-1) in animal models of sepsis to reduce the inflammatory response (13, 18, 19) as well as inhibit cardiomyocyte apoptosis (20, 21). In addition, β-blockers can activate the anti-inflammatory effects of cholecystokinin receptors and thus play a role in protecting the function of the intestinal barrier (18). It has also been shown that β-blockers (landiolol) may improve sepsis-induced acute lung injury through the pulmonary endothelin-1 system (22).

This study had several limitations. First, it was a retrospective analysis and hence suffered from potential selection and ascertainment bias; the baseline characteristics were different between the two groups. To cope with these imbalances, we applied a multivariable Cox proportional hazards regression model and propensity score matching to confirm the robustness of our findings; however, we are not confident that estimation bias of the effects can be completely excluded. Second, due to the retrospective nature of this cohort study, the indications for treatment and treatment intervention were not standardized. Therefore, it was difficult to investigate the impact of dosage on mortality in all participants. Third, owing to the lack of data, we were unable to obtain specific sites of infection as well as objective laboratory indicators of the severity of the inflammatory response to sepsis, such as lactate, PCT, and C-reactive protein levels. Therefore, the results should be interpreted with caution. Finally, a prospective randomized controlled double-blind clinical trial is urgently needed to evaluate the potential benefit and safety of β-blockers given in various doses and routes in septic patients based on sepsis criteria to further prove the results.

## Conclusion

This cohort study suggests that β-blocker use is associated with lower in-hospital mortality in patients with sepsis. Further large-scale prospective studies are needed to verify these findings.

## Data availability statement

The datasets presented in this study can be found in online repositories. The names of the repository/repositories and accession number(s) can be found at: https://mimic.mit.edu/.

## Ethics statement

The requirement of ethical approval was waived by the Clinical Research Ethics Committee of the Second Affiliated Hospital of Guangzhou Medical University for the studies involving humans because the MIMIC-IV database has received ethical approval from the institutional review boards (IRBs) at Beth Israel Deaconess Medical Center and Massachusetts Institute of Technology. The studies were conducted in accordance with the local legislation and institutional requirements. The ethics committee/institutional review board also waived the requirement of written informed consent for participation from the participants or the participants’ legal guardians/next of kin because the database does not contain protected health information.

## Author contributions

QY: Data curation, Methodology, Software, Writing – review & editing. TK: Data curation, Methodology, Software, Writing – original draft. ZB: Data curation, Writing – original draft. SY: Data curation, Writing – original draft. XC: Software, Writing – original draft. JZ: Methodology, Writing – original draft. XX: Writing – review & editing. DW: Supervision, Writing – review & editing. ZZ: Project administration, Writing – review & editing.
