# Supplementary material for: Association between the β-blocker use and patients with sepsis: a cohort study
Source: Front Med (Lausanne). 2023 Oct 26;10:1272871. doi: 10.3389/fmed.2023.1272871 (PMC10641384; doi:10.3389/fmed.2023.1272871)
Supplement: Supplementary file 1 [file Table_1.doc]

| **Patient characteristic** | **Total (n =37923)** | **Non-β-blockers （n=27194）** | **β-blockers （n=10729）** | **P-value** |
| --- | --- | --- | --- | --- |
| Male, n (%) | 20370 (53.7) | 14203 (52.2) | 6167 (57.5) | < 0.001 |
| Age(years) | 65.0 ± 15.9 | 64.0 ± 16.5 | 67.6 ± 14.0 | < 0.001 |
| Vital Signs | | | | |
| Heart rate(bpm) | 105.0 ± 30.7 | 104.5 ± 30.6 | 106.3 ± 30.7 | < 0.001 |
| MAP(mmHg) | 61.0 (51.0, 96.0) | 61.0 (50.0, 94.0) | 62.0 (52.0, 99.0) | < 0.001 |
| Temperature(℃) | 36.4 ± 1.1 | 36.4 ± 1.1 | 36.4 ± 1.1 | 0.859 |
| Laboratory tests | | | | |
| Glucose(mg/dl) | 135.0 (107.0, 167.0) | 134.0 (106.0, 167.0) | 138.0 (110.0, 168.0) | < 0.001 |
| Hemoglobin(g/L) | 10.7 ± 2.2 | 10.6 ± 2.2 | 10.7 ± 2.1 | < 0.001 |
| Platelets(×1012) | 197.6 ± 99.9 | 198.9 ± 100.6 | 194.3 ± 98.0 | < 0.001 |
| WBC(×109) | 13.4 ± 10.3 | 13.4 ± 10.8 | 13.6 ± 8.7 | 0.173 |
| BUN(mg/dl) | 23.0 (15.0, 36.0) | 24.0 (15.0, 37.0) | 23.0 (15.0, 35.0) | 0.986 |
| Creatinine(mg/dl) | 1.2 (0.8, 1.8) | 1.2 (0.8, 1.8) | 1.2 (0.8, 1.7) | 0.003 |
| Bicarbonate(mmol/L) | 23.5 ± 5.6 | 23.4 ± 5.7 | 23.7 ± 5.1 | < 0.001 |
| Potassium(mmol/L) | 4.1 ± 0.7 | 4.1 ± 0.7 | 4.1 ± 0.7 | 0.337 |
| Chloride(mmol/L) | 104.5 ± 6.9 | 104.4 ± 7.0 | 104.9 ± 6.4 | < 0.001 |
| Comorbidity disease,n(%) | | | | |
| Peripheral vascular disease | 326 ( 0.9) | 135 (0.5) | 191 (1.8) | < 0.001 |
| Peptic ulcer disease | 248 ( 0.7) | 157 (0.6) | 91 (0.8) | 0.003 |
| Diabetes | 3371 ( 8.9) | 2046 (7.5) | 1325 (12.3) | < 0.001 |
| Statin use | 5995 (15.8) | 2889 (10.6) | 3106 (28.9) | < 0.001 |
| Severity of illness | | | | |
| Charlson comorbidity index | 3.3 ± 1.6 | 3.2 ± 1.6 | 3.6 ± 1.5 | < 0.001 |
| SOFA score | 6.3 ± 3.1 | 6.3 ± 3.2 | 6.2 ± 2.8 | 0.08 |
| Acute physiology score | 52.4 ± 25.6 | 52.6 ± 26.2 | 51.7 ± 24.2 | 0.001 |
| Apache score | 65.3 ± 26.8 | 65.3 ± 27.5 | 65.3 ± 24.9 | 0.929 |
| In-hospital-mortality,n(%) | 5294(13.6) | 4264(15.2) | 1030(9.6) | < 0.001 |

eTab1.Baseline characteristics of the included patients in eICU database.
