# Supplementary material for: Association between the β-blocker use and patients with sepsis: a cohort study
Source: Front Med (Lausanne). 2023 Oct 26;10:1272871. doi: 10.3389/fmed.2023.1272871 (PMC10641384; doi:10.3389/fmed.2023.1272871)
Supplement: Supplementary file 2 [file Table_2.doc]

eTab2.Association between β-blocker use and in-hospital mortality in the eICU database

| Analysis | in-hospital mortality | P-value |
| --- | --- | --- |
| No.of events/no.of patients at risk(%) |  |  |
| total | 5134/37923(13.6) |  |
| Non-β-blockers | 4264/28119 (15.1) |  |
| β-blockers | 1030/10729(9.6) |  |
| Crude analysis-hazard ratio(95%CI) | 0.49 (0.46,0.52) | < 0.001 |
| Multivariable-hazard ratio(95%CI)^a^ | 0.49 (0.46,0.53) | < 0.001 |
| with matching^b^ | 0.5 (0.46,0.54) | < 0.001 |
| Adjust for propensity score^c^ | 0.49 (0.46,0.52) | < 0.001 |
| Weighted.IPTW^d^ | 0.48 (0.45,0.52) | < 0.001 |

^a^ Hazard ratio from the multivariable Cox proportional model adjusted for all covariates (table1

^b^ Hazard ratio from a multivariate Cox proportional hazards model with the same strata and covariates matched according to the propensity score. The analysis included 10,283 patients (10,283 who received β-blockers and 10,283 who did not).

^c^ Hazard ratio from a multivariable Cox proportional hazards model with the same strata and covariates, with additional adjustment for the propensity score.

^d^ Primary analysis with a hazard ratio from the multivariable Cox proportional hazards model with the same strata and covariates with inverse probability weighting according to the propensity score.
