# Supplementary figures and images for: Association between the β-blocker use and patients with sepsis: a cohort study
Source: Front Med (Lausanne). 2023 Oct 26;10:1272871. doi: 10.3389/fmed.2023.1272871 (PMC10641384; doi:10.3389/fmed.2023.1272871)

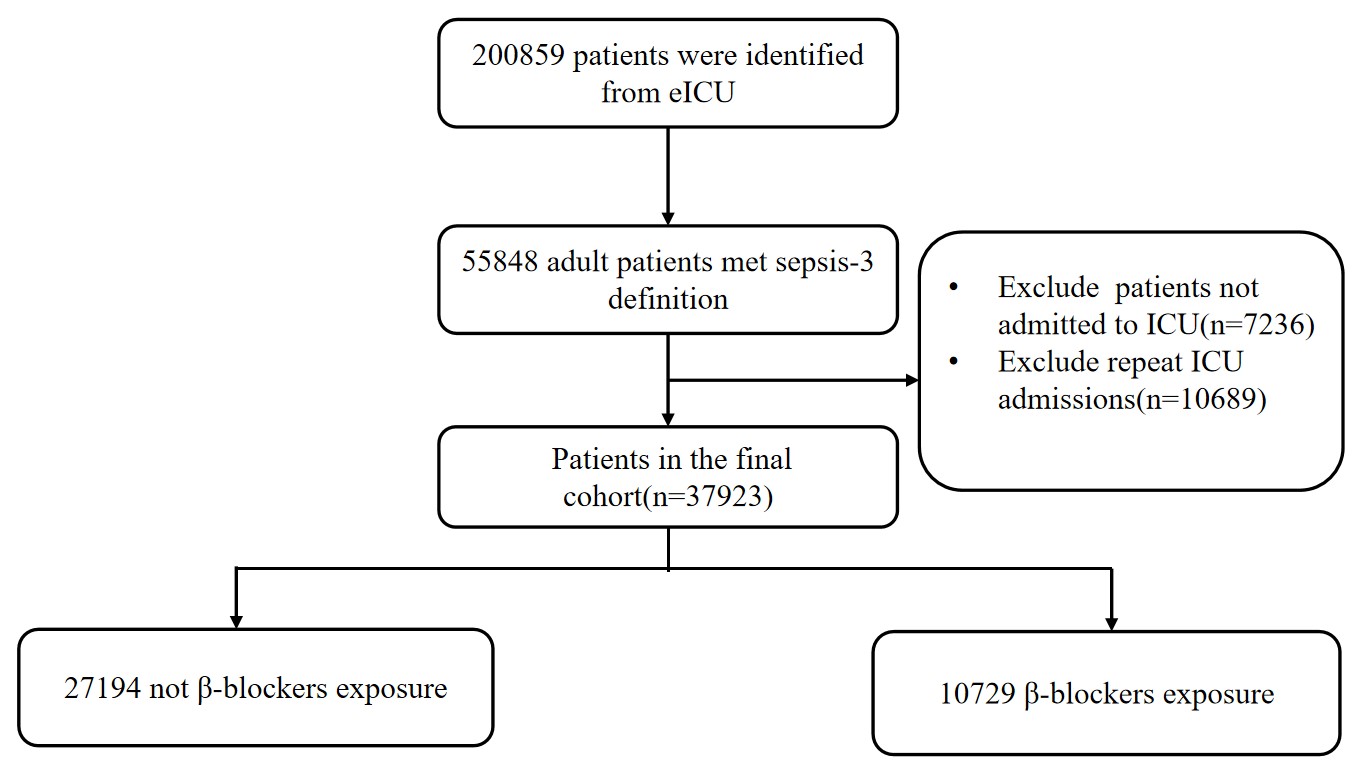


eFig1. The flow chart of the study(eICU database)

Supplement: Supplementary file 3 [file Data_Sheet_1.doc]
